# Minority Participation in Health Research—Facts and Fiction

**DOI:** 10.1371/journal.pmed.0030040

**Published:** 2005-12-06

It is widely believed that racial and ethnic minority groups, especially in the US, are less willing to participate in health research than non-minority groups. According to this view, minority groups' comparative unwillingness to participate is due to a lack of trust in health research and health researchers, which traces to past abuses, particularly the Tuskegee Syphilis Study. Conducted from 1932–1972, the US government–funded Tuskegee study examined the natural course of syphilis. The participants, 399 African-American men in the late stages of syphilis, were enrolled and offered free medical care but kept in the dark about the nature of their illness and the purpose of the study. Participants were told that they were being treated for “bad blood,” but the doctors had no intention of curing them and even withheld penicillin treatment when it became available. When the experiment was finally ended—after public outcry following exposure in the media—28 of the men had died directly of syphilis, 100 were suffering from related complications, 40 of their wives had become infected, and 19 of their children had been born with congenital syphilis.

Given that the study was not halted until 1972, it would not be surprising if its memory influenced the attitudes of minority individuals toward health research today. This is a potential problem, because it is essential that participants in health research are as diverse as the population whose health should be improved as a result of the research. (And in the US today, one in five people is from a minority group). Only representative participation ensures that the outcomes of health research can be generalized to a diverse population.

Is participation in health research representative of the population? A number of studies suggest that in the US it is not; minority groups are often under-represented in US research studies. But what are the reasons? Are minority groups less willing to participate, or are they given fewer opportunities to participate? Answering this question is vital for efforts to increase minority participation in health research. Should these efforts focus on changing minority attitudes or on removing potential barriers to participation, such as whether minorities are adequately informed of research opportunities?

In a systematic review in this month's *PLoS Medicine*, David Wendler and colleagues assessed whether individuals from minority groups who were eligible and invited to participate in health research were indeed less likely to consent to participate than non-minority individuals. The authors identified 20 health research studies that reported consent rates by race or ethnicity. Eighteen were single-site studies conducted exclusively in the US or multi-site studies conducted primarily or exclusively in the US. The 20 studies collectively report the enrollment decisions of over 70,000 individuals for a broad range of health research studies.

For the three non-intervention studies, African-Americans had a non-significantly lower overall consent rate than non-Hispanic whites, while Hispanics had a non-significantly higher overall consent rate than non-Hispanic whites. For the ten intervention studies, African Americans' overall consent rate was non-significantly higher than that of non-Hispanic whites, while Hispanics had a statistically significant higher overall consent rate than non-Hispanic whites. For the seven surgery trials, minorities as a group had a non-significantly higher overall consent rate than non-Hispanic whites.

Although Wendler and colleagues found only small differences in consent rates by race or ethnicity, they found substantial differences by race or ethnicity in the number of individuals invited to participate. For example, one study of medical versus surgical management of angina offered enrollment to 2,065 non-Hispanic whites but to only 30 individuals from minority groups.

This study, Wendler and colleagues say, “suggests that racial and ethnic minorities are as willing as non-Hispanic whites to participate in health research.” Indeed for some kinds of studies, minority individuals seem more willing to enroll than non-minority individuals. The authors acknowledge their study's limitations, particularly the fact that most individuals were from the US and the willingness of minority groups outside the US to participate in health research may be very different. Other important questions not addressed by this study are what motivates people to participate in health research, and whether motives differ between majority and minority groups. Despite these and other remaining questions, the results of this study suggest that efforts to increase minority participation in health research should concentrate on ensuring better access to health research for all groups, rather than on changing the attitudes of minority groups.

